# Adaptive tuning of infrared emission using VO_2_ thin films

**DOI:** 10.1038/s41598-020-68334-2

**Published:** 2020-07-14

**Authors:** M. C. Larciprete, M. Centini, S. Paoloni, I. Fratoddi, S. A. Dereshgi, K. Tang, J. Wu, K. Aydin

**Affiliations:** 1 Dipartimento di Scienze di Base ed Applicate per l’Ingegneria, Sapienza Università di Roma, Via Antonio Scarpa 16, 00161 Rome, Italy; 2 Dipartimento di Ingegneria Industriale, Università degli Studi di Roma Tor Vergata, Via del Politecnico 1, 00133 Rome, Italy; 3 Dipartimento di Chimica, Sapienza Università di Roma, 00185 Rome, Italy; 4 Department of Electrical and Computer Engineering, Northwestern University, Evanston, IL 60208 USA; 5 Department of Materials Science and Engineering, University of California, Berkeley, CA 94720 USA; 6 Materials Sciences Division, Lawrence Berkeley National Laboratory, Berkeley, CA 94720 USA

**Keywords:** Metamaterials, Materials science, Phase transitions and critical phenomena

## Abstract

Phase-transition materials provide exciting opportunities for controlling optical properties of photonic devices dynamically. Here, we systematically investigate the infrared emission from a thin film of vanadium dioxide (VO_2_). We experimentally demonstrate that such thin films are promising candidates to tune and control the thermal radiation of an underlying hot body with different emissivity features. In particular, we studied two different heat sources with completely different emissivity features, i.e. a black body-like and a mirror-like heated body. The infrared emission characteristics were investigated in the 3.5–5.1 μm spectral range using the infrared thermography technique which included heating the sample, and then cooling back. Experimental results were theoretically analyzed by modelling the VO_2_ film as a metamaterial for a temperature range close to its critical temperature. Our systematic study reveals that VO_2_ thin films with just one layer 80 nm thick has the potential to develop completely different dynamic tuning of infrared radiation, enabling both black-body emission suppression and as well as mirror emissivity boosting, in the same single layer device. Understanding the dynamics and effects of thermal tuning on infrared emission will benefit wide range of infrared technologies including thermal emitters, sensors, active IR filters and detectors.

## Introduction

The possibility to tune the spectral features of a device, triggered by a thermal, electrical or optical stimulation, paves the way for many applications such as perfect infrared absorbers^1–4^, smart intelligent window coatings^5,6^, thermal rectification devices^7^ or diodes/rectifiers^8^. Thermochromic materials, such as niobium dioxide (NbO_2_), vanadium sesquioxide (V_2_O_3_), and vanadium dioxide (VO_2_) undergo an abrupt phase transition from semiconductor to metallic state at a specific phase transition temperature^9–11^. This phase change transition, from insulating monoclinic phase into a metallic tetragonal (rutile) phase, is accompanied by drastic changes in electrical, optical and magnetic properties as a function of the temperature. Among them, VO_2_ is widely employed since it pertains the lowest phase transition temperature, T_c_ = 341 K (68 °C)^12^.

The strong and reproducible changes in optical properties due to phase transition, have generated a burgeoning amount of theoretical and experimental studies focusing on the thermal emissivity variation activated by temperature in VO_2_^13,14^ in the IR^15^ and THz radiation ranges^16^.

The thermal hysteresis intrinsic to the phase transition has been widely investigated and was found to be affected by several factors such as the film deposition temperature^17^, the use of different substrates^18–20^, the wavelength range^21^, the introduction of metallic dopants^22^ and the dimensions of metallic grains^23^. In particular, if metallic grain size is smaller than the wavelength of excitation, diffraction effects result in enhanced transmitted signal. When temperature increases, the grain sizes of expanding metallic phases increase, which in turn yield to enhanced diffraction that is manifested in the red-shift of the wavelength of maximum transmittance^23^.

Extremely large tuning of infrared absorption has been shown in a perfect absorber composed by just one thin layer of VO_2_ onto sapphire substrate, where interference effects allowed absorption switching from 20 to 99.5 at 11.6 micron^1^. Efficient terahertz modulation performance was also experimentally demonstrated, in terms of strong modulation depth of transmittance (83%), using 120 nm thick films^16^.

Furthermore, VO_2_ layers are also widely employed for the realization of tunable and reconfigurable metamaterials. Bi-tunable transmission and absorption characteristics have been demonstrated ny hBN/graphene/hBN heterostructures combined with Ge gratings on one side and a hybrid VO_2_/Au grating on the other side^24^. Specifically, a spectral absorption peak attributed to excitation of magnetic resonance can be excited below phase transition temperature, while above the phase transition temperature this magnetic resonance vanishes in the metallic state^24^. In Ref.^25^ a layer of phase change VO_2_ (470 nm thick) was combined to other layers (150 nm thick TiN layer and a Si film of 320 nm) and to a frequency selected surface (FSS) composed by an array of circular patches of aluminum, to realize a metamaterial thermal emitter^25^. An array of slot nano-antennas with tunable phase response have been designed for electronic beam steering applications, where tuning is induced by a temperature gradient over the VO_2_ layer^26^. More interestingly, metasurfaces of VO_2_^27^ in combination with natural hyperbolic polar materials, such as hBN, enable the tuning, manipulation and/or enhancement of optical phonon modes. Nanopatternened metasurfaces have been proposed to tune polaritonic bands of polar material, where the plasmonic coupling is excited in VO_2_ above phase transition temperature^28^. The complex pattern of polaritons in hBN have been also experimentally imaged using SNOM measurements from a reconfigurable metasurface where hBN has been transferred on top of a single crystal VO_2_ layer^29^.

In the present work, we experimentally investigate infrared emission from a single VO_2_ thin film on sapphire substrate using infrared thermography technique^30,31^. We aim to tune infrared emission characteristics of a hot object that can find use in thermal filtering and infrared camouflage applications. We investigated the emission properties of VO_2_ film in the 3.3–5.1 μm wavelength range. The rationale behind our choice of specific wavelength range is due to the transparency of the Al_2_O_3_ substrate in this particular IR window since the tunable features arise from the interplay between the emission properties of VO_2_ and the heated body under the Al_2_O_3_ substrate. For this purpose, we studied two different configurations where the investigated device is placed in contact either with a *black-body* or with an infrared *mirror-like* heat source. We are interested in evaluating and understanding the effects of the different contributions to the overall IR radiation and eventually quantifying the critical role of VO_2_ as the active tuning material for thermal emission. Moreover, we identify the contributions of the heat source to spectral features like emittance or reflectance that will eventually benefit future device designs.

In this study, we first outline the investigated device and describe two different experimental configurations, along with the IR thermographic measurements. We then experimentally demonstrate the versatile, adaptable IR emission features of the same device under different heating. Finally, we provide a theoretical background for the analysis and interpretations of the obtained experimental data, where the different roles played by VO_2_ device and heat sources are identified and discussed.

## Results

**Figure 1:**
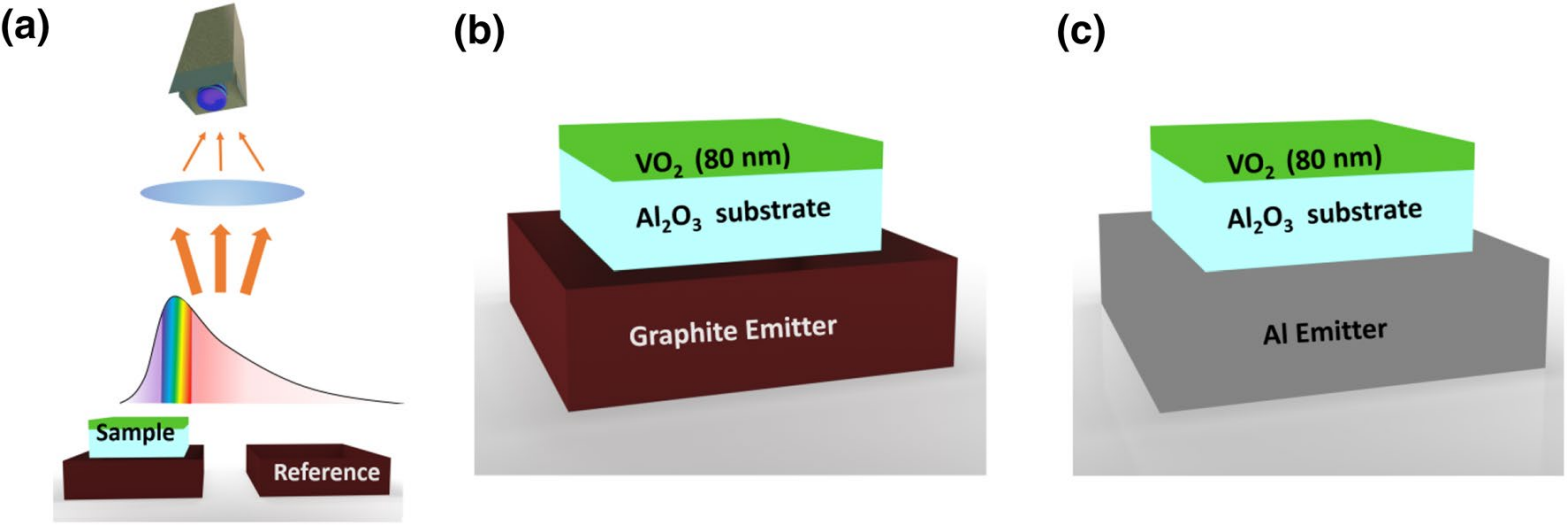
Sketches of investigated experimental configuration and samples. (**a**) Schematic representation of the experimental setup. Sample is placed on (**b**) a high emissivity reference paint (black-body-like) source, (**c**) a low emissivity metal plate (mirror-like) source.

The system under investigation consists of an 80 nm thick VO_2_ film layer (d ~ *λ*/50 at central wavelength *λ* = 4 μm) on c-plane sapphire substrate by pulsed-laser-deposition (PLD), allowing high transmission through the entire operational wavelength range of the infrared camera (3.3–5.1 μm). The experiment as well as the geometry of the investigated configurations is schematically depicted in Fig. 1.

The infrared emitted signal has been characterized by means of infrared thermography according to the ASTM E1933-99a standard method^32^ adopting a reference material with well-known IR emissivity. Specifically, in the non-contact configuration, the sample and a thin layer of a reference medium (black-soot^33^ or graphite^34^) are simultaneously imaged by the IR camera in order to obtain the different IR signals emitted by both of them, i.e. by sample and by a reference black-body respectively.

The measurements on the VO_2_ films have been carried out by recording thermographic images for increasing temperature of the heating plate between temperatures 50 °C and 85 °C and eventually cooling down, with a heating/cooling rate of 1 °C/min^35^. At the end of each temperature run, the recorded thermographic images were processed via a dedicated software developed in MATLAB environment enabling us to select the image pixels corresponding to either the sample or the reference surface and, hence, to obtain the corresponding value of the average emitted power.

**Figure 2:**
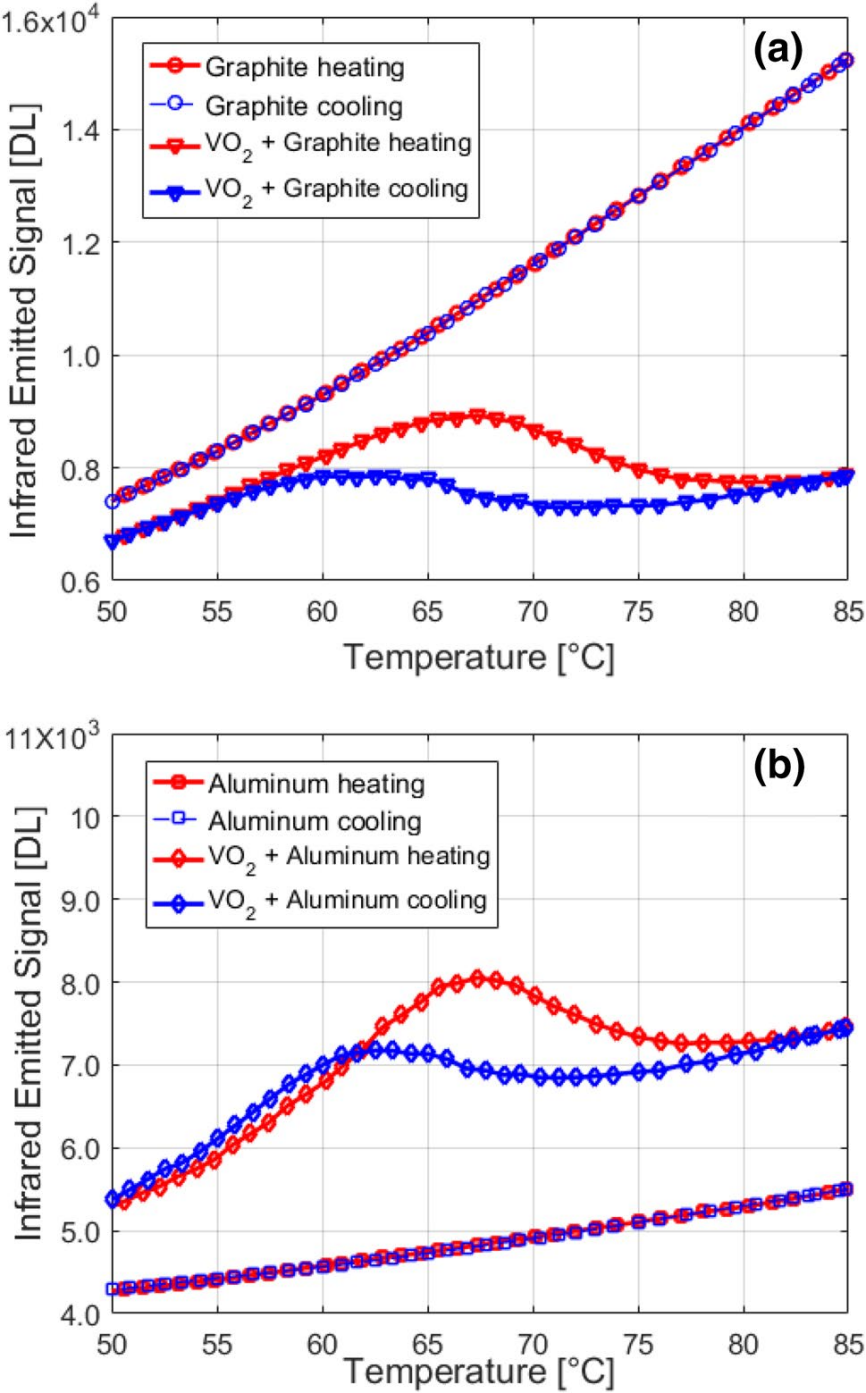
(**a**) Emitted power of the VO_2_-sapphire sample placed onto graphite (triangles) compared to the emitted power from bare graphite (circles). (**b**) Emitted power of the VO_2_-sapphire sample placed onto aluminum (diamonds) compared to the emitted power from bare aluminum (squares). Red curves represent heating process while blue curves hold for cooling down.

The infrared signals measured in the 3.3–5.1 μm range arising from four different cases are plotted as a function of temperature in Fig. 2, including (a) graphite reference and VO_2_ structure over graphite, (b) VO_2_ structure over metallic plate and aluminum reference plate. In this figures, red colors indicate the heating case while the blue lines correspond to measurements during the cooling stage. It is worth noting that the signals coming from the VO_2_ structures clearly display typical hysteresis curve, due to the intrinsic phase transition taking place in the VO_2_ layer, despite its few tens of nm thickness. The signals emitted from the graphite and the aluminum plate expectedly do not show any hysteresis. Indeed, they are the same for both heating and cooling processes.

**Figure 3:**
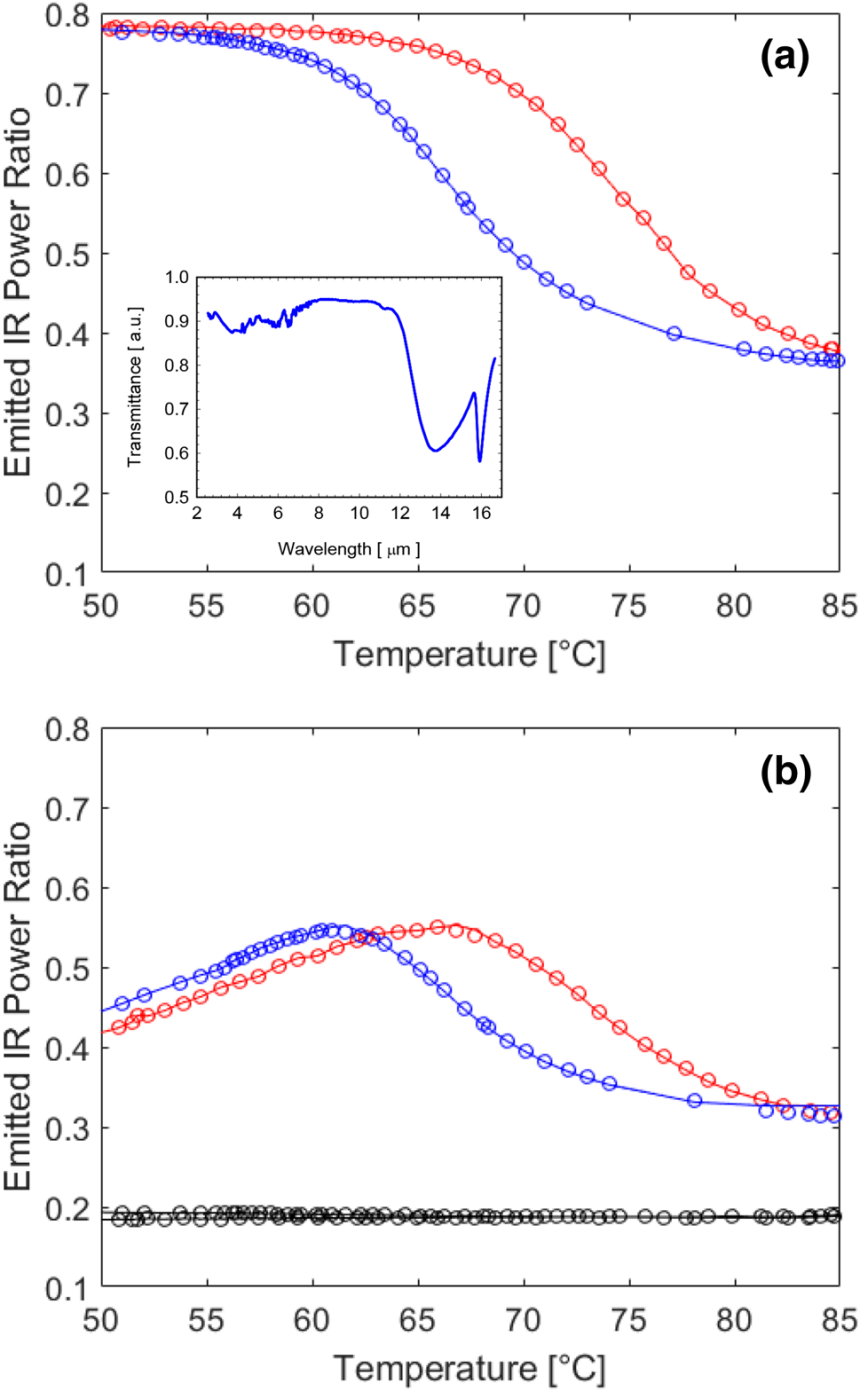
Relative emitted power of the VO_2_-sapphire sample on (**a**) graphite and (**b**) aluminum for heating (red curves) and cooling (blue curves). Emitted signal from bare aluminum (black curve) is also displayed in (**b**). Markers represent experimental data while continuous lines are the theoretically fit curves. Inset: FTIR transmission spectra of the VO_2_-sapphire in the cold phase.

From the obtained experimental data, we computed the ratio of emitted signals with respect to that of emitted by the reference in order to distinguish the contribution of VO_2_ in reshaping the emission characteristics of the device. The profiles of obtained ratios through measuring the two different experimental configurations, are displayed in Fig. 3a,b with markers and the solid curves show simulation results. The overall emitted signal S_sample_ integrated on the camera wavelength range (3.3–5.1 microns) and normalized with respect to the reference perfect blackbody emitter (S_ref_—reference graphite paint) can be interpreted as an average effective relative emissivity of the system in the camera wavelength range *ε*_*eff*_(*T*) according to:

1 $$ \frac{{S_{sample\left( T \right)} }}{{S_{ref\left( T \right)} }} = \frac{{\mathop \smallint \nolimits_{{\lambda_{min} }}^{{\lambda_{max} }} \left( {\frac{{2hc^{2} \varepsilon_{eff} \left( {\lambda ,T} \right)}}{{\lambda^{5} \left( {e^{{\left( {\frac{hc}{{k_{B} \lambda T}}} \right)}} - 1} \right)}}} \right)d\lambda }}{{\mathop \smallint \nolimits_{{\lambda_{min} }}^{{\lambda_{max} }} \left( {\frac{{2hc^{2} \varepsilon_{graphite} \left( {\lambda ,T} \right)}}{{\lambda^{5} \left( {e^{{\left( {\frac{hc}{{k_{B} \lambda T}}} \right)}} - 1} \right)}}} \right)d\lambda }} = \left\langle {\varepsilon_{eff} \left( T \right)} \right\rangle $$

where $\frac{{2hc^{2} }}{{\lambda^{5} \left( {e^{{\left( {\frac{hc}{{k_{B} \lambda T}}} \right)}} - 1} \right)}}$ is the power spectral density per unit area radiated by a perfect black body, *h* is the Planck's constant, *c* is the speed of light in vacuum, *k*_*B*_ is the Boltzmann constant, *λ*_*min*_ and *λ*_*max*_ represent the lower and upper limit of the detected wavelength range, respectively^36^.

The measured effective relative emissivity as defined in Eq. (1) is illustrated as markers in Fig. 3a,b for the configurations sketched in Fig. 1a,b, respectively. We also report, in the inset of Fig. 3a, the Fourier-transform infrared spectroscopy (FTIR) spectrum of the VO_2_ layer on sapphire substrate in its cold phase. It is worth to note that in the investigated wavelength range the sample is mostly transparent, with respect to the long wavelength infrared (LWIR) range where the vibration modes of the substrate significantly decrease the transmittance of the substrate^37^. For this reason, we expect that in our target wavelength range, which encompasses medium wavelength infrared (MWIR) band, the system composed by the heat source and the device will display different features depending on the choice of the heat source and the principal layer of interest, VO_2_, as opposed to LWIR, where the properties are strongly dominated by the sapphire substrate emissivity.

Figure 3 suggests that the overall system behaves differently if the sample is placed on top of a high emitting material (graphite—Fig. 1a) or a low emitting material (aluminum plate—Fig. 1b). Figure 3a infers that when the VO_2_ structure is placed over and the black-body (BB)-like emitter, the infrared radiation arising from the VO_2_/Sapphire/BB system switches from high to low value as temperature is increased above *T*_*c*_, with a large dynamic range of about 0.4. On the other hand, if the VO_2_/Sapphire sample is placed in direct contact with a low emissivity mirror-like source, a polished aluminum plate whose measured relative emissivity is shown in the same Fig. 3b (black dotted curve), the overall resulting infrared radiation is not monotonically decreasing with the temperature. It is also worth to note that there is a maximum emissivity wavelength across the VO_2_ transition temperature in Fig. 3b, leading to measured higher temperature of the overall system. In the first case (Fig. 3a), high emissivity of graphite is gradually shielded during the transition of VO_2_ from insulator to metallic phase. Contrarily, in the second case (Fig. 3b), during the phase transition, the emissivity of the VO_2_ layer gradually increases and the overall forward emissivity of the sample is enhanced by the presence of the mirror-like plate which reflects the backward emission. When the phase transition is complete, the sample is almost opaque and the contribution of the bottom layer is negligible. Supporting this claim, is the fact that for high temperatures above *T*_*c*_, the average relative emissivity is almost the same for both systems and approximatively equal to the emissivity of the VO_2_ layer only. Finally, the temperatures corresponding to the maximum negative slope of the emissivity both for heating and cooling processes (i.e. critical temperatures, *T*_*c*_) were extracted from the experimental data. We performed numerical evaluation of the derivatives of the emissivity curves. Critical temperatures, corresponding to the points of minimum for the derivatives, were found to be *T*_*c*_(heating) = 73 °C and a *T*_*c*_(cooling) = 66 °C, in agreement with literature results^19^.

**Figure 4:**
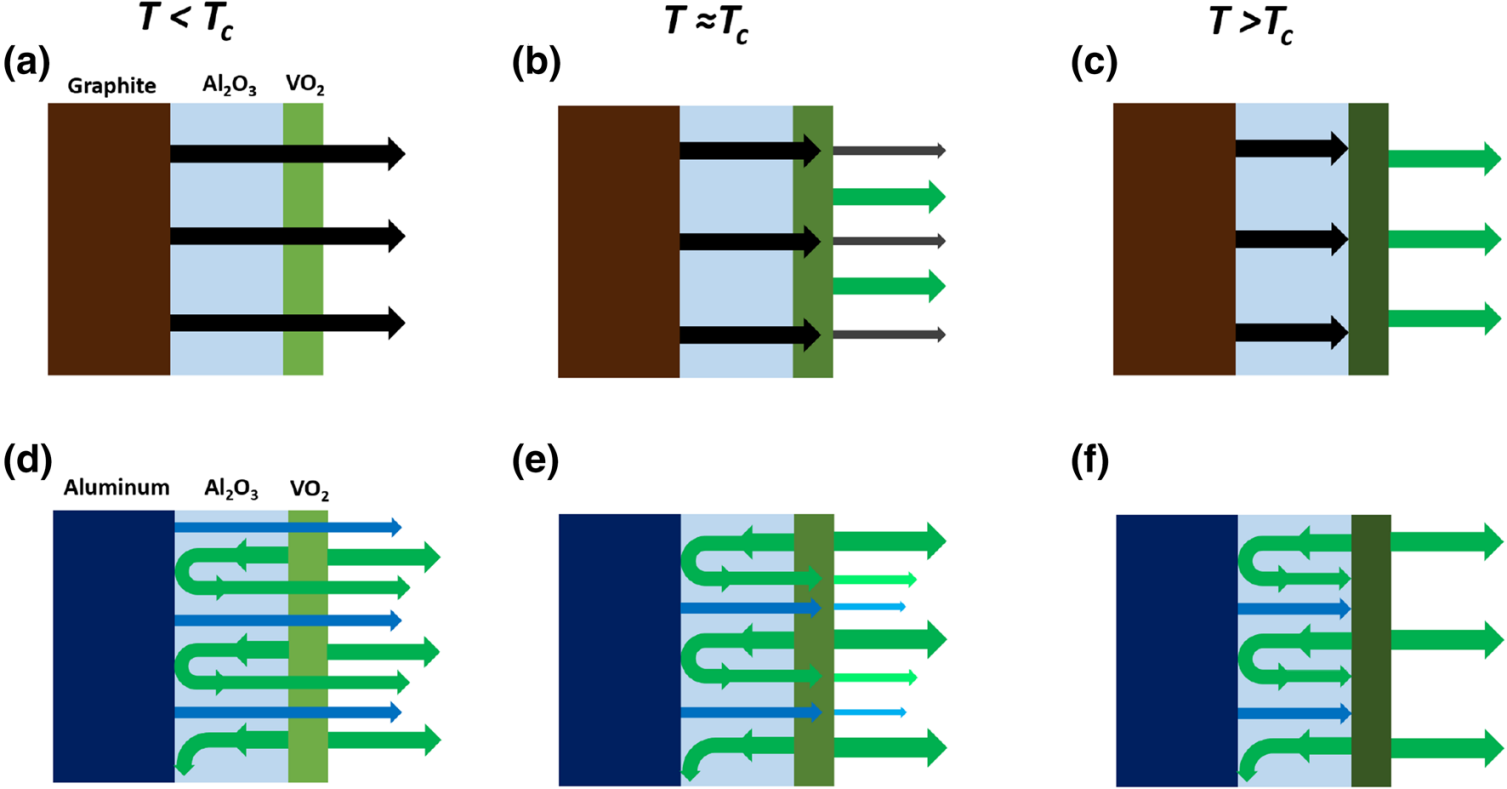
Schematic illustration of different emissivity term contributions at different temperature ranges below and above the phase change temperature for the sapphire-VO_2_ sample on (**a**–**c**) graphite emitter and (**d**–**f**) aluminium emitter. Green arrows hold for the infrared radiation emitted by VO_2_ sample. Black arrows and blue arrows display the infrared radiation emitted by graphite (**a**–**c**) and alumina (**d**–**f**), respectively.

We provide a qualitative description of the different behaviors in Fig. 4a,b in order to schematically illustrate our claims. In the next section, we will analyze and discuss the dynamics of the effect of phase transition on emissivity and we will quantitatively show that differences between the two systems are related to the different reflectivity of the bottom layers.

## Discussion

According to the fundamental principles of quantum mechanics, every hot object at its given absolute temperature emits electromagnetic radiation. The physical phenomenon governing thermal emission from hot objects has been widely studied for over a century and is summarized by Planck’s law, stating that a so called black body (BB), i.e. an ideal body that perfectly absorbs over the entire electromagnetic spectrum for all incident angles, emits electromagnetic radiation with a spectrum determined by its temperature, *T*. However, for real bodies, the relative thermal emissivity designates the ability of an object to generate and radiate heat compared to a BB and is determined by its optical absorptivity^38^. Within the context of this model, we interpreted the experimental data obtained when the device consisting of VO_2_ layer onto sapphire substrate is in contact with two heat sources exhibiting totally different emissivity features, namely a black body (graphite paint) and an infrared mirror (aluminium plate). From the experimental results we extract an effective average emissivity of the system which takes into account both the VO_2_ device and the heat source.

Microscopically, in the VO_2_ thin film, the phase transition is progressively occurring as the temperature is increased^39,40^. Here, the metallic phase emerges as randomly distributed nanoscale inclusions into a semiconductor matrix, giving rise to percolation effects as the filling factor is increased from *f* = 0 (full semiconductor state) to *f* = 1, at the end of the phase transition. Given the fact that the VO_2_ film is much thinner than the wavelength of interest, this phenomenon can be macroscopically modelled as a metamaterial emitter using effective medium theory^41^. Thus, we calculated refractive index and extinction coefficient across the entire semiconductor to metal phase transition, ranging from the semiconducting VO_2_ (*f* = 0; below phase transition temperature) to the metallic VO_2_ (*f* = 1, above phase transition temperature). The refractive index values of VO_2_ in the semiconductor and metallic states were taken from Ref.^42^.

In order to describe the temperature behavior of VO_2_, we employed a model based on Maxwell Garnett mixing formulas for ellipsoidal inclusions reported in reference^21,43,44^. Furthermore, it can be easily shown that for discoidal inclusions the effective permittivity evaluated with the Maxwell Garnett approach produces the same results as the Looyenga mixing formula, (i.e. a static solution for a planar mixture) successfully adopted in^45^. This model facilitates the calculation of the relative permittivity of an effective medium, composed by a dispersion of inclusions into a matrix^46^. For such an effective medium, the relative permittivity is found to dramatically vary along the three orthogonal directions. As a general feature, the electrical permittivity dyadic of such a medium assumes the following form:

2 $$ \mathop \varepsilon \limits^{ = } = \left( {\begin{array}{*{20}c} {\varepsilon_{\alpha } } & 0 & 0 \\ 0 & {\varepsilon_{\beta } } & 0 \\ 0 & 0 & {\varepsilon_{\gamma } } \\ \end{array} } \right) $$

where complex permittivity component along *α*-, *β*- and *γ*-axes are calculated using:

3 $$ \varepsilon_{eff,j} = \varepsilon_{S} + f\frac{{\varepsilon_{S} \left( {\varepsilon_{M} - \varepsilon_{S} } \right)}}{{\varepsilon_{S} + \left( {1 - f} \right)L_{j} \left( {\varepsilon_{M} - \varepsilon_{S} } \right)}}. $$

Here, *ε*_*S*_ and *ε*_*M*_ stand for the relative permittivities of the host matrix (semiconductor VO_2_) and the inclusions (metallic VO_2_), respectively, and the inclusion ratio is determined by the filling factor, *f ,* while the term *L*_*j*_ (*j* = *α*, *β* and *γ*) represents the depolarization factors^45^. Specifically, the three depolarization factors are determined by the ratios between the ellipsoidal inclusions’ axes and, for any shape of inclusions, should always fulfill the requirement *L*_*α*_ + *L*_*β*_ + *L*_*γ*_ = *1*. Using this model, we can describe different configurations, depending on inclusions’ shape and orientation with respect to coordinate axes. According to^17,47^, the optical properties of VO_2_ thin films across the phase transition can be modeled by considering 2D inclusions. In the case under examination the out of plane dimension is limited by the film thickness which is only 80 nm. We thus chose disk like inclusions shape as detailed in^48^. Following these considerations, we fix *L*_*α*_ = 1 (along disk thickness) and *L*_*β*_ = *Lγ* = 0 (along disk diameters).

Once the directional dielectric permittivity components are calculated as a function of the filling factor, it is possible to retrieve refractive indices and extinction coefficients along the corresponding axes, using the well-established relations $Re\left( \varepsilon \right) = n^{2} - k^{2}$ and $Im\left( \varepsilon \right) = 2nk$.

The investigated 1D structure is particularly suitable to be computed rapidly by transfer matrix method (TMM) techniques, while some care must be taken for the complex refractive index. In these simulations, instead of hot or room temperature phases of VO_2_, we consider an equivalent layer, having the calculated effective medium properties, and retrieve its optical transmission and reflection on axis normal to the film surface, at increasing filling factor values. Let *T*(*ω*) and *R*(*ω*) be the real transmittance and reflectance for the thin film, representing the ratios of transmitted and reflected optical power with respect to an incident electromagnetic field, normalized to unity. If absorption is present in the layer, we may define the real absorbance by:

4 $$ A\left( \omega \right) = 1 - R\left( \omega \right) - T\left( \omega \right) $$

which is essentially an expression of energy conservation. The transmittance through both Al and graphite is negligible. The connection between absorbance and emittance is drawn using Kirchoff’s second law which states that the ratio of thermal emittance *ε*(*ω*) to the absorbance *A*(*ω*) is a constant independent of the nature of the material and that its value is unity^49,50^. As a result, the thermal spectral power of a thin film structure from Kirchhoff’s law is given by *ε*(*ω*) = *A*(*ω*).

According to the qualitative interpretation sketched in Fig. 4, a numerical model for the overall effective emissivity of the system should include three terms contributing to the infrared signal *S*_*sample*_(*T*) detected by the infrared camera. The first term represents the infrared radiation emitted by the VO_2_ film ($\varepsilon_{VO_2} \left( {\lambda , T} \right)$) over sapphire substrate, which is assumed to be transparent in the considered wavelength range (green arrows, Fig. 4a–f). The second term takes into account the infrared radiation which is emitted by the heater, *ε*_*heater*_, (black-body-black arrows Fig. 4a–c-or mirror-like heater-blue arrows Fig. 4d–f) and is transmitted by the VO_2_ layer ($T_{VO_2} \left( {\lambda , T} \right)$). Finally, the infrared reflectance (1 − *ε*_*heater*_) of the heater must be considered, i.e. reflection from bottom interface. If the heater is highly reflective, as for metallic surfaces, the infrared radiation emitted by the VO_2_ is back reflected by the heater and then is forward transmitted by the VO_2_ itself, giving the third term of the equation. Thus, the effective relative emissivity of the system can be modeled as:

5 $$ \varepsilon_{eff} \left( {\lambda , T} \right) = \varepsilon_{VO_2} \left( {\lambda , T} \right) + \varepsilon_{heater} \cdot T_{VO_2} \left( {\lambda , T} \right) + \left( {1 - \varepsilon_{heater} } \right) \cdot T_{VO_2} \left( {\lambda , T} \right) \cdot \varepsilon_{VO_2} \left( {\lambda , T} \right) $$

**Figure 5:**
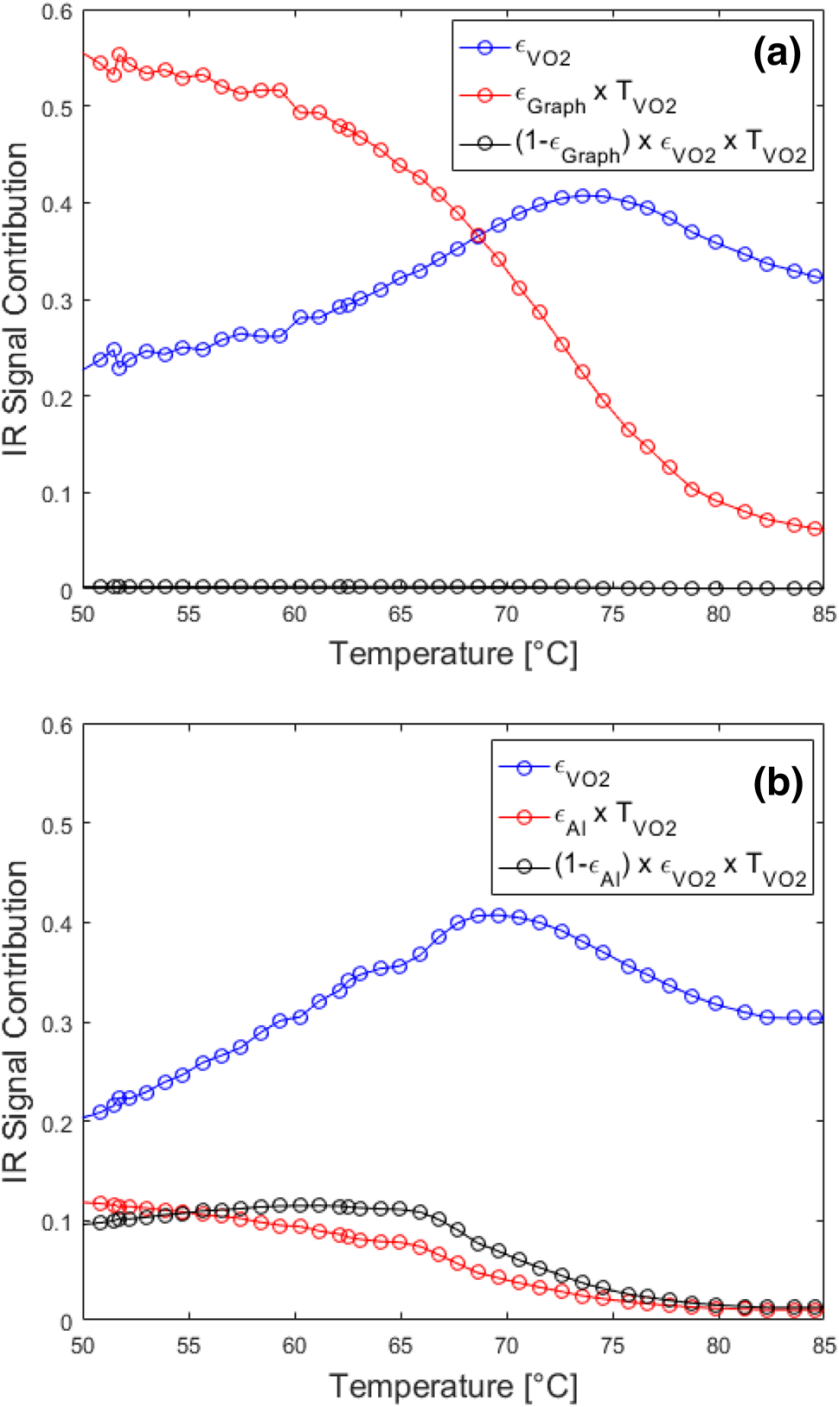
Calculated individual contribution of each term of Eq. (5) to the total effective emissivity for both configurations of the VO_2_-sapphire sample on (**a**) graphite and (**b**) aluminium for heating.

The obtained experimental curves, reported with symbols in Fig. 3a,b, have been reconstructed, according to Eq. (5) by applying a least squares fitting procedure where the filling factor of metallic phase was the only fitting parameter. Specifically, for each temperature point, during both the heating and cooling processes, once the filling factor is fixed, the resulting effective dielectric permittivity (see Eq. (3) ) allows to calculate the spectral features such as reflectance, transmittance (and thus absorbance) using TMM method. Concerning ε_heater_ we assumed a constant emissivity value for the graphite paint of 0.98 and a constant emissivity for the polished aluminum plate of 0.2. The three contributions to the effective relative emissivity of the system [see Eq. (5)] were then integrated in the Planck power spectrum calculation [Eq. (1)] in the operational wavelength range of the IR camera (λ_min_ = 3.3 μm and λ_max_ = 5.1 μm). The obtained fitting curves, for the two different experimental configurations, are also displayed in Fig. 3a,b with solid lines, respectively. Finally, in Fig. 5 we schematically report the individual contributions of each of the term of Eq. (5) to the total effective emissivity for both configurations (sample on top of graphite—Fig. 5a, or aluminum, Fig. 5b). As expected, in the first case in Fig. 5a, only two first terms from Eq. (5) are dominant: the signal emitted from graphite and transmitted by the sample (red curve) and the signal emitted by the sample (blue curve). It is clear from this figure that the signal from the high emissivity source is strongly attenuated as the temperature increases beyond *T*_*c*_, where the overall emission from the VO_2_ sample becomes dominant. A similar behavior is obtained for *T* > *T*_*c*_ when the sample is placed on top of the aluminum plate. However, we show (Fig. 5b) that in this case the emitted signal form the aluminum plate and transmitted through the sample (red curve) is lower than the signal emitted by the sample (blue curve) for all the temperature values which is due to the low emissivity of aluminum mirror. Moreover, unlike graphite case, a non-negligible role is played by the backward emission form the sample reflected by the aluminum surface (black curve) which again can be attributed to the strong mirroring behavior of aluminum layer. These results highlight that the emission can be enhanced or suppressed on demand by a sub-wavelength VO_2_ thin film. The changes in spectral features introduced by the phase change material can be exploited to tune the overall infrared emissivity.

Finally, a fine tuning of the hysteresis loop could be achieved by properly selecting the VO_2_ thickness. More specifically, in Ref.^19^ is shown that an enlargement of the hysteresis loop is observed due to the size dispersion of the crystallites and a progressive increase of the thermal hysteresis width is observed with the decrease of the thickness of the VO_2_, that can be related to the formation of VO_2_ crystallites with smaller domains^19^.

## Conclusions

In summary, we investigated the IR radiation from a device consisting of a thin VO_2_ film deposited onto sapphire substrate. Two different configurations were studied, where the thermochromic device was placed in contact either with a *black-body* or with an infrared *mirror-like* heat source by means of infrared thermography in the 3.5–5.1 μm spectral range, under heating regime. For both configurations, despite of the extremely different properties of the heating sources, the spectral changes introduced by the phase change material results in active tuning of infrared emitted radiation. The obtained experimental results show that the active coating of VO_2_ can suppress the IR radiation from underlying black body or, alternatively, can enhance the IR radiation emitted by a low emittance material (metallic plate, mirror) at around *T*_*c*_, i.e. in proximity of the anomalous absorbance peak. The two different configurations highlight the on-demand infrared emission features that can be achieved using the same device, in terms of both active layer and substrate, depending on the nature of the heat source.

Despite the emissivity of the heated source, above the transition temperature of the VO_2_, the emissivity is kept at a constant value. This result achieved with a film as thin as 80 nm reveals the camouflage efficiency of VO_2_ for both high emitting and low emitting sources highlighting a very versatile solution for different operating environments.

Finally, by tuning and optimizing VO_2_ layer thickness, similar structures can be designed to get a highly transmissive structure that can turn into a low emitting structure, when temperature is increased above VO_2_ phase transition temperature, incorporating Fabry–Perot condition to the wavelength of interest.

## Methods

### Sample realization

The VO_2_ thin film was deposited in 5 mTorr O_2_ environment at 550 °C substrate temperature, and the PLD laser energy was set at 321 mJ with 5 Hz pulse frequency. A post-deposition anneal at 550 °C for 30 min was performed in the same 5 mTorr O_2_ environment.

### Infrared thermographic measurements

The samples are placed on a stabilized heat source, which is provided with a Peltier modulus. Above the heater, a layer made of graphite paint (Bonderite L-GP 386 Acheson) characterized by a well-known IR emissivity value^51^ is deposited next to the sample location. Finally, two control thermocouples are placed in contact with the surface of the heater in order to monitor the actual temperature evolution of sample and reference during measurements.

An IR camera CEDIP Jade MWIR operating in the mid-infrared wavelength range (3.5–5.1 μm) has been adopted to measure the amount of emitted IR radiation. The radiometric imaging system is based on a 320 × 240-pixel InSb focal plane array, with a pixel pitch of 30 μm and a thermal sensitivity corresponding to a noise equivalent temperature difference (NETD) of 25 mK at 300 K. The IR camera system is accompanied by a manual focusing lens with a focal length of 25 mm. During the measurements, the camera axis was held slightly tilted with respect to the sample normal in order to prevent the spurious IR radiation reflected from the sample to be detected by the camera. In addition, the IR camera software allows us to account for the background temperature, so that IR radiation contribution to the measured signal arising from the spurious environmental radiation can be compensated.

## Data Availability

The data that support the plots within this paper and other findings of this study are available from the corresponding author upon reasonable request.
